# Single-molecule real-time sequencing of the full-length transcriptome of *Halophila beccarii*

**DOI:** 10.1038/s41598-022-20988-w

**Published:** 2022-09-30

**Authors:** Siting Chen, Guanglong Qiu

**Affiliations:** Guangxi Key Lab of Mangrove Conservation and Utilization, Guangxi Mangrove Research Center, Guangxi Academy of Sciences, Beihai, 536007 Guangxi China

**Keywords:** Molecular biology, Plant sciences

## Abstract

Ecologically, *Halophila beccarii* Asch. is considered as a colonizing or a pioneer seagrass species and a “tiny but mighty” seagrass species, since it may recover quickly from disturbance generally. The use of transcriptome technology can provide a better understanding of the physiological processes of seagrasses. To date, little is known about the genome and transcriptome information of *H. beccarii*. In this study, we used single molecule real-time (SMRT) sequencing to obtain full-length transcriptome data and characterize the transcriptome structure. A total of 11,773 of the 15,348 transcripts were successfully annotated in seven databases. In addition, 1573 long non-coding RNAs, 8402 simple sequence repeats and 2567 transcription factors were predicted in all the transcripts. A GO analysis showed that 5843 transcripts were divided into three categories, including biological process (BP), cellular component (CC) and molecular function (MF). In these three categories, metabolic process (1603 transcripts), protein-containing complex (515 transcripts) and binding (3233 transcripts) were the primary terms in BP, CC, and MF, respectively. The major types of transcription factors were involved in MYB-related and NF-YB families. To the best of our knowledge, this is the first report of the transcriptome of *H. beccarii* using SMRT sequencing technology.

## Introduction

Seagrasses are flowering monocotyledonous plants that have fully adapted to the lifecycle of being completely submerged in the ocean^1^. In evolutionary history, four independent seagrass lineages evolved from terrestrial species to living in the marine environment, forming independent but often convergent adaptation strategies^2,3^. Seagrass meadows are one of the most widely distributed coastal ecosystems on earth^2^, and they have important ecosystem service functions. For example, seagrass meadows provide important food sources and habitats for marine animals, such as green turtles and dugongs^4,5^. Simultaneously, seagrass meadows are key places for carbon storage in the biosphere^6^.

However, seagrass meadow ecosystems are facing multiple threats, including eutrophication, sediment cover, species invasion, human fishery activities, pathogen invasion, global warming, ocean acidification, and typhoons, and the area of seagrass meadows has declined sharply^7,8^. Research on the mechanism of degradation and the protection of seagrass meadows has become an internationally recognized research hotspot. Seagrass conservation is urgent. Thus, we used RNA-Seq to obtain the full-length transcriptome to provide some genetic resources for the conservation of seagrasses.

*Halophila beccarii* Asch. is a typical intertidal seagrass. It belongs to one of the two oldest lineages of all the seagrasses and is known as the "living dinosaur"^9^. Biologically, it has the characteristics of "old age," including a small shape, quick growth, monoecious status, pistils that ripen first, low genetic diversity, and the coexistence of annual and perennial life histories^10^. Ecologically, it is considered as a colonizing or a pioneer seagrass species and a “tiny but mighty” seagrass species, since it may recover quickly from disturbance generally^9^. *H. beccarii* is one of 10 current species of seagrass that are at risk for extinction^11^. Owing to its limited distribution range, quick population turnover, small shape, and tendency to easily be covered by sediments, *H. beccarii* and its importance are not well known^12^. Although it has high research and ecological value as an important species, there is a dearth of genomic and transcriptome information.

Currently, the transcriptome research of seagrass primarily depends on technologies, such as RNA-Seq, expression sequence tags (EST), and DNA microarrays. Revealing the mechanism of sexual reproductive of seagrass and the differences in transcriptome between different tissues at the molecular level will help to understand the reproductive, life history characteristics and genetic basis of seagrass, but there are currently few studies in this field. A comparative study of the differential gene expression between the leaves and male and female flower tissues of *Posidonia oceanica* showed that the genes related to photosynthesis and metabolic processes were upregulated in the leaves, while the genes related to cell wall tissue, growth, and external capsule structure were significantly upregulated in the flowers. In addition, the genes that are enriched in the female flower tissues are related to photosynthesis, protein chromophore connection and chlorophyll biosynthesis, indicating their contribution to sexual reproduction^13^. In addition to *P. oceanica*, the molecular mechanism of sexual reproduction and the differences of transcriptome between the tissues of other seagrasses also merit urgent study. Simultaneously, the biological functions of non-coding RNA (ncRNA), which has biological functions, such as gene splicing, RNA modification, protein transport and housekeeping^14^ merit further study. How seagrass responds to light stress through the regulation of gene expression is one of the research hotspots in transcriptomics^15–18^. Similarly, temperature stress is also one of the research hotspots^19–21^. In addition, the regulatory mechanism of gene expression in seagrass in response to high salinity^22–24^, heavy metals^25,26^, and CO_2_ stress^27^ has also been reported. Studies have shown that different genotypes of seagrass populations have varying responses to stress and recovery to the same environmental stress^28–31^.

RNA sequencing (RNA-Seq) has become a powerful method to generate most sequence data and cDNA sequences. It can provide new and comprehensive information for gene research^32^. For decades, many studies on RNA-Seq have been utilized to understand gene expression and potential molecular mechanisms, particularly for non-model species that lack reference genome^33–36^. RNA-Seq helps to study mRNA splicing, gene expression, and candidate gene screening, but it provides limited information on gene structure and full-length sequences^37^. In addition, the extent of alternative splicing (AS) and transcriptome diversity remains largely unknown because of its short read length^38^. Recently, single molecule real-time (SMRT) sequencing technology has completely changed the limitations of short reading sequences without fragmentation and post sequencing assembly. In addition, it provides accurate full-length transcripts with an average sequence reading of up to 50 kb^39,40^. Therefore, SMRT sequencing, as an effective tool, has been widely and successfully used in the annotation and analysis of full-length transcripts of plants, such as sugar beet (*Beta vulgaris*)^40^, *Zostera japonica*^41^and *Z. muelleri*^26^.

In this study, SMRT sequencing was used to produce full-length transcripts of *H. beccarii*. The transcriptome annotation and structure were then analyzed. The simple sequence repeats (SSRs) of *H. beccarii* were obtained by our SMRT sequencing. The results of this study will provide a valuable and comprehensive genetic resource for further study on the gene function and biological regulatory mechanism of *H. beccarii*. The intraspecific genetic diversity of *H. beccarii* with the characteristics of "colonizing species" was relatively low^42,43^. The SSRs obtained by our SMRT sequencing can be used to further analyze the genetic diversity of *H. beccarii*, which is an endangered species. *H. beccarii* is often described as a "colonizing species" because it can rapidly expand its population with the help of asexual reproduction, i.e., the horizontal growth of rhizomes, and it can also establish a new population through sexual reproduction, i.e., the diffusion of seeds^44,45^. We analyzed differences in the transcriptome in the leaves and rhizomes of *H. beccarii*. These data can provide a molecular basis for further study on the physiology and the conditions that result in the endangered status of *H. beccarii*.

## Results

### Full-length transcript data output

**Figure 1:**
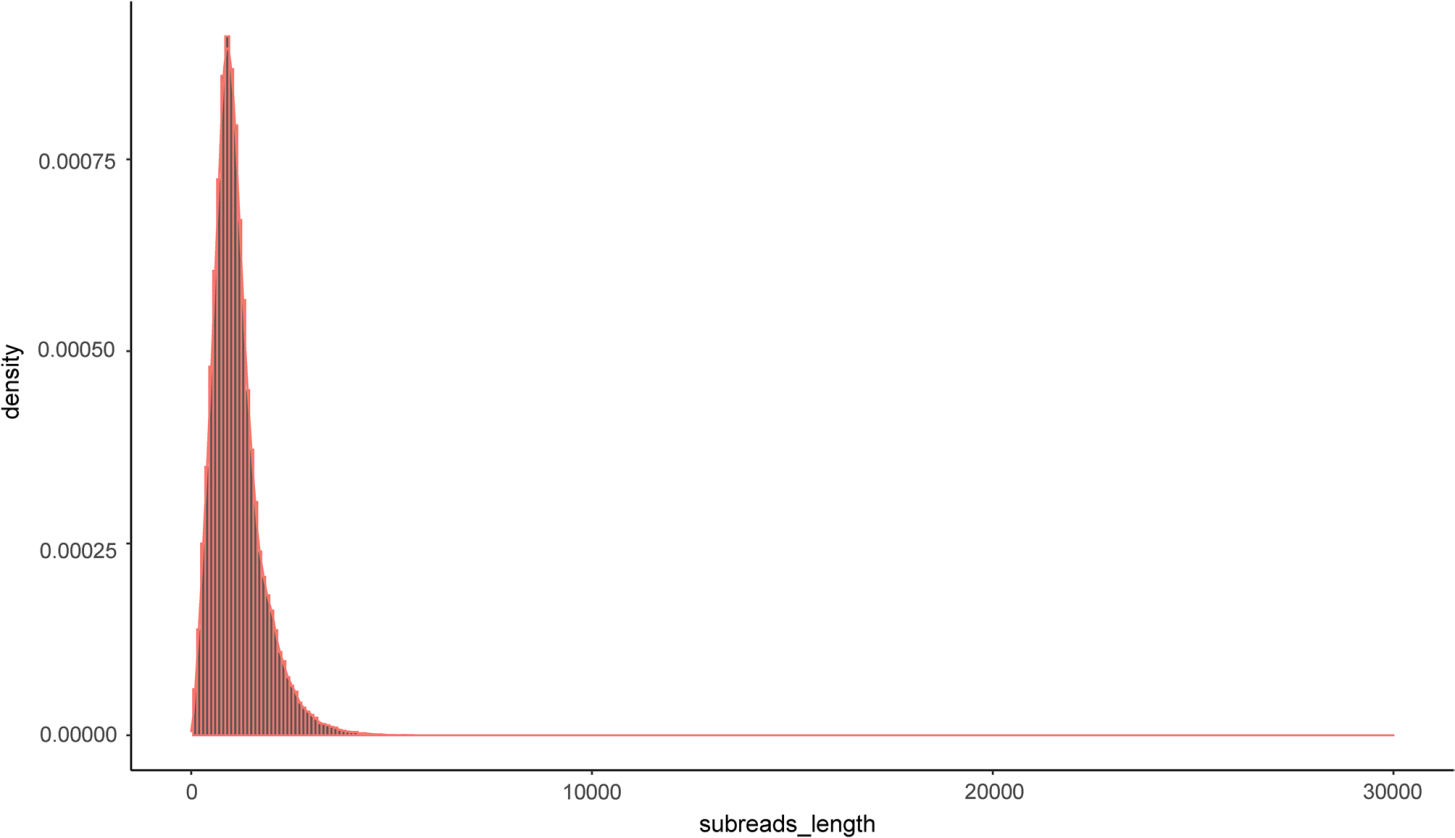
FLNC length distribution. FNLC, full-length non-concatemer.

The plant materials of *H. beccarii* were collected in Shajing, Qinzhou, Guangxi, China. The sampling site, outside mangrove forests, was covered by dense *H. beccarii* (Fig. S1). A total of 325 Mb read bases of circular consensus sequences (CCSs) were obtained. A total of 272,028 CCSs were acquired with a mean length of 1194 bp (Table 1). A subsequent analysis revealed that 213,301 full-length non-concatemer sequence (FLNC) reads were identified (Fig. 1). After clustering, consensus isoforms were generated with an average read length of 1011 bp, which resulted in 21,264 polished high-quality isoforms (Table 1). Finally, 16,303 non-redundant transcripts were generated.

**Table 1:** Summary of PacBio SMRT sequencing in *Halophila beccarii* Asch.

Category	Dataset
Read bases of CCS	325,064,946
Number of CCS	272,028
Mean Read Length of CCS	1194
Number of full-length non-chimeric reads	213,301
Full-length non-chimeric percentage (FLNC%)	78.41%
Average consensus isoforms read length	1011
Number of polished high-quality isoforms	21,264

*CCS* circular consensus; *SMRT* single nucleotide real-time.

### ORF and transcription factors (TFs) prediction

**Figure 2:**
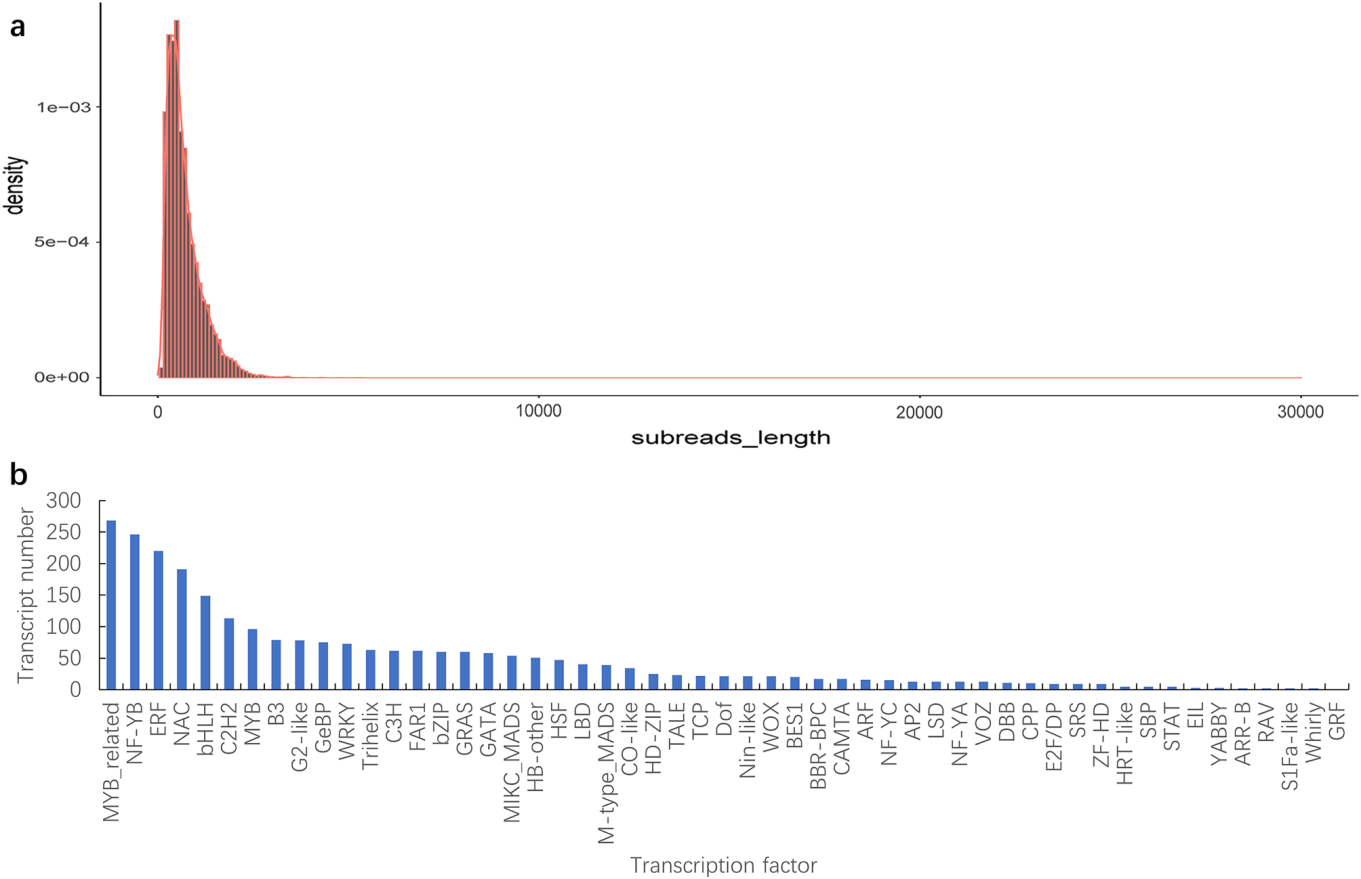
(**a**) Length distribution of CDS and (**b**) Type distribution of TFs. CDS, coding sequence; TFs, transcription factors.

A total of 15,348 open reading frames (ORFs) were identified. As shown in Fig. 2a, CDS < 1 kb was dominant (12,204, 79.52%). A total of 2567 TFs were detected, and the major types were involved in MYB-related and NF-YB families (Fig. 2b).

### Functional annotation of transcripts

A total of 15,348 identified transcripts were scanned against seven databases (Table S1). The annotation rate was 5843 (38%) in Gene Ontology (GO), 5517 (35%) in the Kyoto Encyclopedia of Genes and Genomes (KEGG), 6951 (45%) in EuKaryotic Orthologous Groups (KOG), 11,632 (75%) in RefSeq non-redundant proteins (nr), 9865 (64%) in Pfam, 9612 (62%) in SWISS-PROT and 11,652 (75%) in TrEMBL. A total of 15,348 identified transcripts of *H. beccarii* were BLASTed with the protein sequences of seagrass species *Z. muelleri,* and approximately 10,000 transcripts can be aligned to the protein sequence of *Z. muelleri*. The high similarity of annotation with *Z. muelleri* shows that our assembly quality was sufficient.

To understand the biological function of the *H. beccarii* transcriptome, a KEGG pathway analysis was conducted. The results showed that 5517 (35%) transcripts were enriched in 271 signaling pathways. The primary pathways were protein processing in endoplasmic reticulum (468, 8.48%) and ribosome (433, 7.85%), followed by carbon metabolism (292, 5.29%), biosynthesis of amino acids (254, 4.60%) and glycolysis/gluconeogenesis (232, 4.21%) (Table 2).

**Table 2:** The top 20 mapped pathways annotated by the KEGG database.

Pathways	Pathway ID	Gene number	Percentage
Protein processing in endoplasmic reticulum	ko04141	468	8.48%
Ribosome	ko03010	433	7.85%
Carbon metabolism	ko01200	292	5.29%
Biosynthesis of amino acids	ko01230	254	4.60%
Glycolysis / Gluconeogenesis	ko00010	232	4.21%
Endocytosis	ko04144	162	2.94%
Spliceosome	ko03040	161	2.92%
Oxidative phosphorylation	ko00190	153	2.77%
Plant-pathogen interaction	ko04626	141	2.56%
RNA transport	ko03013	137	2.48%
Epstein-Barr virus infection	ko05169	136	2.47%
Phagosome	ko04145	128	2.32%
Carbon fixation in photosynthetic organisms	ko00710	116	2.10%
Flavonoid biosynthesis	ko00941	115	2.08%
Phenylpropanoid biosynthesis	ko00940	110	1.99%
PI3K-Akt signaling pathway	ko04151	108	1.96%
Plant hormone signal transduction	ko04075	107	1.94%
Starch and sucrose metabolism	ko00500	106	1.92%
Arginine and proline metabolism	ko00330	104	1.89%
Photosynthesis—antenna proteins	ko00196	102	1.85%

*KEGG*, Kyoto Encyclopedia of Genes and Genomes.

**Figure 3:**
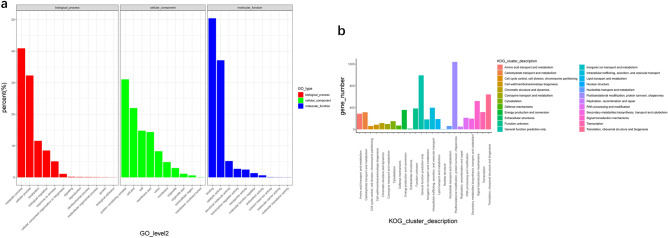
(**a**) GO annotation and (**b**) KOG annotation of *Halophila. beccarii* transcriptome. GO, Gene Ontology; KOG, EuKaryotic Orthologous Group.

To classify the function of all the full-length transcripts, GO annotation was performed (Fig. 3a). A GO analysis showed that 5843 transcripts were divided into three categories, including biological process (BP), cellular component (CC) and molecular function (MF). In these three categories, metabolic process (1603 transcripts), protein-containing complex (515 transcripts) and binding (3233 transcripts) were the primary terms in BP, CC, and MF, respectively. The KOG classification was also performed to further study the function of the *H. beccarii* transcripts. A KOG analysis showed that 6951 transcripts were grouped into 24 categories. The dominant subclasses were posttranslational modification, protein turnover, and chaperone (1240, 17.84%), followed by general function prediction only (993, 14.28%) and translation, ribosomal structure and biogenesis (642, 9.24%) (Fig. 3b).

### lncRNA prediction

**Figure 4:**
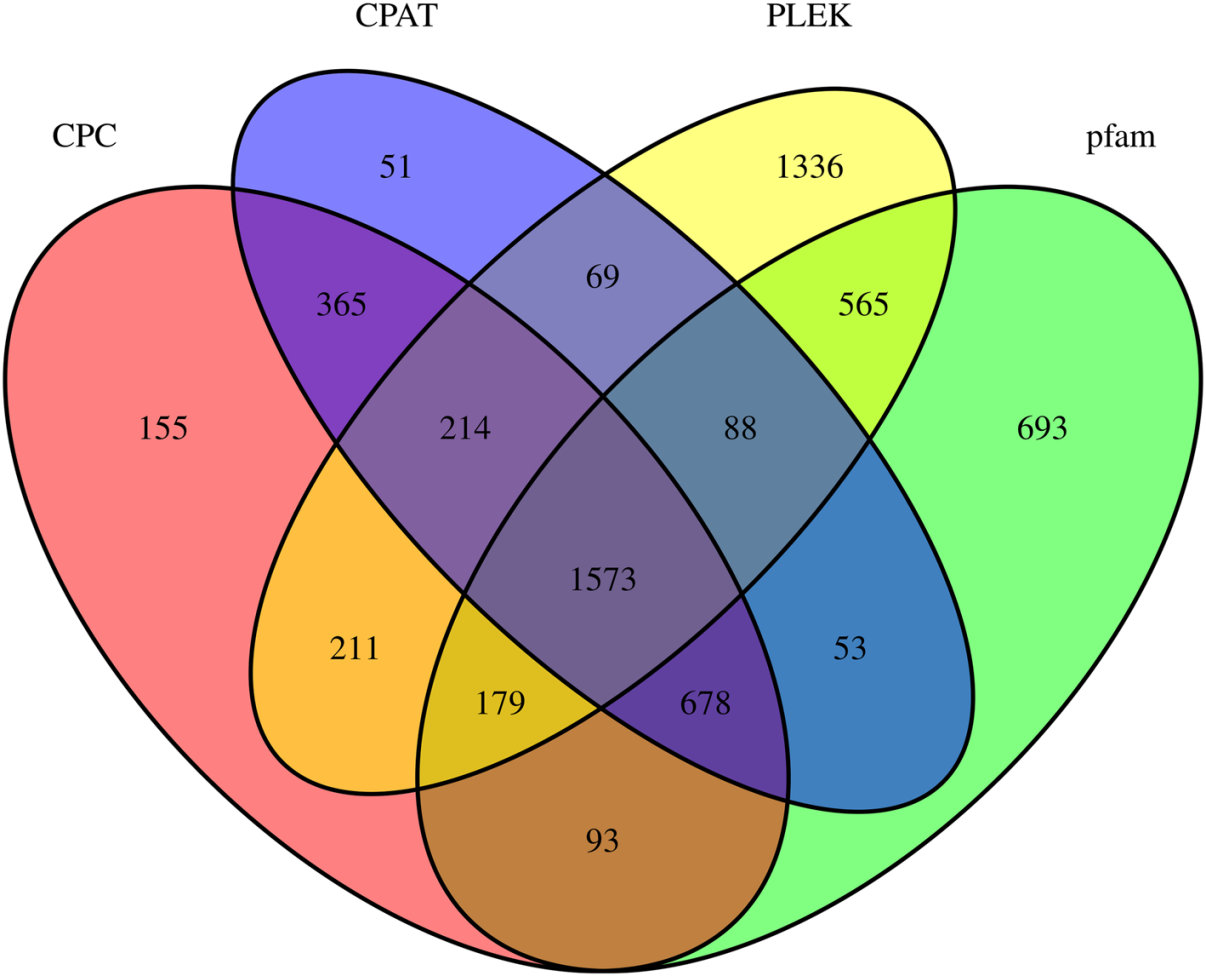
Candidate lncRNAs identified by PLEK, CPC2.0, CPAT and Pfam. lncRNAs, long non-coding RNAs.

Four computational tools were combined and used to predict the number of lncRNAs, including the PLEK, CPC2.0, CPAT and Pfam databases. The results revealed that 4235, 3468, 3091, and 3922 lncRNAs were obtained in the PLEK, CPC2.0, CPAT, and Pfam databases, respectively. Among them, 1573 lncRNAs were common in the four approaches (Fig. 4). The lncRNAs detected by the four methods are shown in Table S2.

### SSR prediction

A total of 8402 SSRs were identified in 6822 sequences that contained SSRs. Among these transcripts, 1366 contained more than one SSR. Furthermore, the most abundant were mononucleotides (3766, 55.20%), followed by trinucleotides (1190, 17.44%). The frequency of di-, tetra-, penta- and hexanucleotides was 7.15% (601), 0.95% (80), 0.30% (25), 0.32% (27), respectively (Table S3). Table S4 lists all the SSRs and their corresponding primers.

### Reference sequence alignment

We aligned the original sequencing reads to the full-length transcript to subsequently quantify the level of gene transcription, reconstruct the transcripts, and discover new genes. The aligned statistical results are shown in Table S5.

### Organ-specific expression analysis

**Figure 5:**
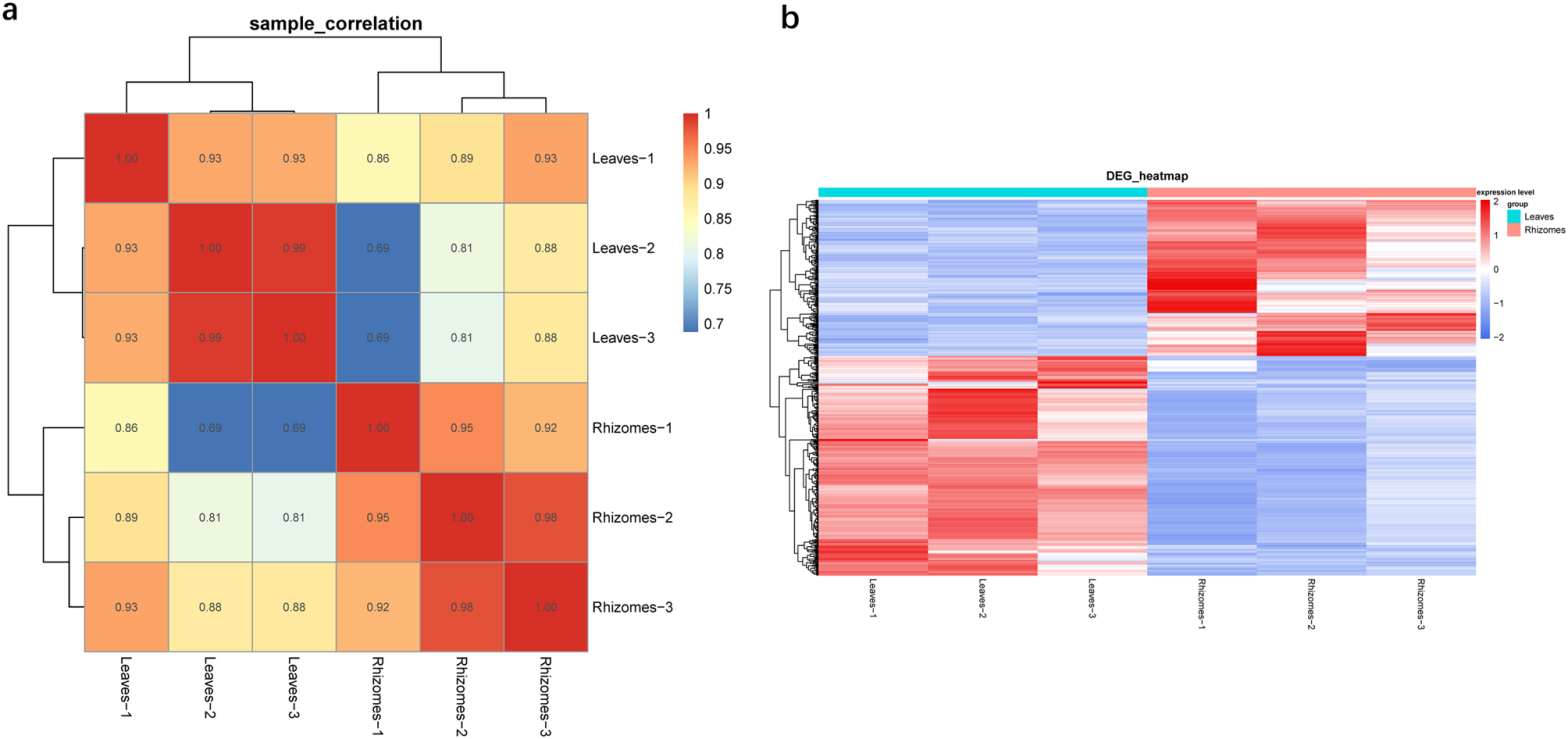
(**a**) Schematic diagram of the correlation between samples and (**b**) heatmap of a hierarchical cluster analysis of the patterns of gene expression in the leaves and rhizomes of *Halophila. beccarii.*

We screened 189 upregulated genes and 266 downregulated genes in the rhizomes to compare them with the leaves (Table S6). The top 20 differentially expressed genes (DEGs) in the rhizomes compared with leaves are shown in Table 3. All the biological replications appeared to be clustered according to the sample type (leaf and rhizome tissue), and there was no significant difference between the sample and replication relationship (Fig. 5a). Accordingly, a hierarchical cluster analysis of gene expression (Fig. 5b) revealed clear patterns of differential expression between the leaf and rhizome tissues.

**Table 3:** The top 20 DEGs in the rhizomes compared with the leaves.

DEGs	Fold change	p value
Alcohol dehydrogenase 2 (ADH2)	2.461847516	1.45E−28
Protein DMR6-like oxygenase 2 (DLO2)	2.870885051	3.59E−15
Naringenin,2-oxoglutarate 3-dioxygenase	2.016622651	5.86E−14
L-type lectin-domain containing receptor kinase IV.1	2.884287285	1.17E−11
Probable ATP synthase	0.384616453	4.69E−11
Transcription factor bHLH123	2.451808363	6.07E−11
Transcription factor MYB4	2.417753479	2.43E−10
Cellulose synthase-like protein	2.121630125	6.65E−10
Probable cinnamyl alcohol dehydrogenase 1 (CAD1)	3.378190526	1.25E−09
Zinc finger protein 10 (ZFP10)	2.428728573	1.73E−09
PGR5-like protein 1A (PGRL1A)	0.338663384	1.83E−09
Probable apyrase 1 (APY1)	2.023614949	2.04E−09
Heavy metal-associated isoprenylated plant protein 39 (HIPP39)	2.212188767	2.24E−09
Respiratory burst oxidase homolog protein C (RBOHC)	2.113301068	3.83E−09
Cytochrome b5	2.232792949	5.67E−09
NifU-like protein 3 (NIFU3)	0.198341181	1.23E−08
Serine/threonine-protein kinase SAPK6	2.173676296	2.00E−08
RNA replication polyprotein	3.324898518	2.12E−08
Ferredoxin-dependent glutamate synthase	0.418434506	3.78E−08
Nudix hydrolase 1	2.015395898	7.84E−08

*DEGs*, differentially expressed genes.

### Characterization of the significant functional properties of DEGs in the leaves and rhizomes of *H. beccarii*

**Figure 6:**
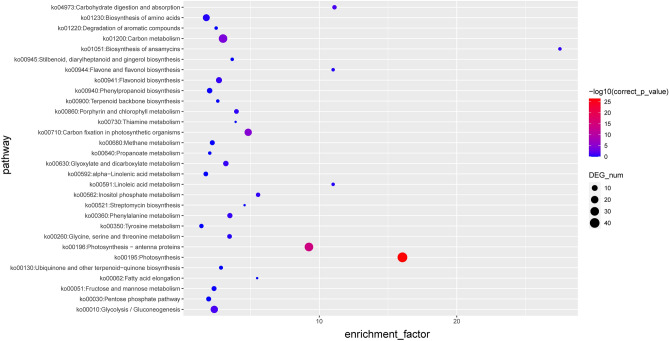
KEGG enrichment analysis Bubble Diagram of upregulated gene expression in the leaf tissues of *Halophila beccarii*. The size of circle reflects the number of differentially expressed genes. KEGG, Kyoto Encyclopedia of Genes and Genomes.

The first three KEGG rich items in the leaves are related to photosynthesis, photosynthesis-antenna proteins, and carbon fixation in photosynthetic organisms according to p value (Fig. 6 and Table S7). The rich set of photosynthetic genes (Table S8) includes genes that encode the oxygen-evolving complex (*PsbR*, *PsbO*, *PsbP* and *PsbQ*), photosynthetic system I (*PsaD*, *PsaE*, *PsaF*, *PsaH*, *PsaK*, *PsaL* and *PsaN*), PSII-LHCII supercomplexes (*PsbW*) and the cytochrome b_6f._ complex (Rieske [Fe-S] protein).

Furthermore, 91 TFs were detected in the DEGs, and the major types were involved in the ERF and M-type_MADS families (Fig. S2).

## Discussion

In this study, SMRT sequencing was used to produce full-length transcripts of *H. beccarii*. The transcriptome annotation and structure were then analyzed. The SSRs obtained by SMRT sequencing can be used to further analyze the genetic diversity of *H. beccarii*. We analyzed the differences in the transcriptome of leaves and rhizomes of *H. beccarii*. These data can provide a molecular basis for further study on the physiology and the conditions that result in the endangered status of *H. beccarii*.

In the face of increasing stress, the resources of *H. beccarii* throughout the world are continuously declining. *H. beccarii* is considered to be a "tiny but mighty" seagrass because it can often recover quickly after interference. Obtaining the full-length transcriptome and understanding the structure of genes for *H. beccarii* is a primary step to study gene functions that are highly significant yet remain unknown.

SMRT sequencing provides new knowledge of full-length sequences, which has been proven to be helpful in performing gene annotation and interpreting gene functions, particularly for species that lack reference genomes^38,46^. In this study, we obtained 272,028 CCSs and identified 213,301 FLNC, which then yielded 16,557 corrected isoforms with an average read length of 1041 bp.

It is now recognized that lncRNAs act as local regulators and mediate the expression of adjacent genes through RNA protein interactions^47–49^. lncRNAs are involved in plant growth and development^50^, the regulation of flowering, reproductive development^51–53^ and stress responses^54^. In recent years, the rapid development of third-generation sequencing technology characterized by the sequencing of single molecules enables the direct sequencing of lncRNAs and the detection of modifications on these molecules^55^. A large number of lncRNAs have been identified from Arabidopsis^56^, rice (*Oryza sativa*)^57^, *Gossypium australe*^58^ and other species. However, there have been no previous reports of lncRNAs in *H. beccarii*. In our study, 1573 common lncRNAs were predicted by four types of software, which will promote the further functional study of these lncRNAs in the *H. beccarii* transcriptome.

MYB transcription factors are one of the largest families of plant transcription factors (TFs). The MYB TF family refers to a class of TFs that contain an MYB domain, which is a class of highly conserved DNA binding domains. The MYB domain is a highly conserved peptide composed of approximately 50–52 amino acids as a repeat. MYB TFs are widely involved in biological functions in plants, particularly in the response to stress^59^. Increasing amounts of evidence support the concept that MYBs are important TFs that improve biological and abiotic stress resistance^60,61^. Recent studies have shown that nuclear factor Y (NF-Y) is an important family of plant TFs. There have been many reports on the involvement of NF-Y transcription factors in the regulation of plant growth, development, and defense against stress. The levels of expression of *NF-YB3* and *NF-YB2* increased when flowering was induced in Arabidopsis, and these genes were involved in the regulation of plant flowering characteristics^62^. Some NF-Y subunits are involved in the regulation of nodule formation, initial flowering, blue light response, and the chloroplast development of legume plants owing to their effects on the transcription of downstream genes^63–65^. These studies show that the NF-Y family TFs are widely involved in plant growth, development, and stress response biological processes. In this study, 2567 TFs were detected in *H. beccarii*, and the major types were involved in MYB-related and NF-YB families (Fig. 2b). This provides basic data for the in-depth study of the biological processes of growth, development, and stress responses of *H. beccarii*.

Photosynthetic oxygen release originates from the light reaction of photosynthesis, which is performed by the oxygen-evolving complex (OEC) located on the inner side of the thylakoid lumen^66,67^. In plants and algae, OEC is composed of an Mn_4_O_5_Ca cluster of photosystem II (PSII) and its ligands and the four external proteins PsbO (33 kD), PsbP (23 kD), PsbQ (17 kD) and PsbR (10 kD)^66,68–71^. The four extrinsic proteins of OEC are encoded by nuclear genes and play a key role in the release of oxygen^69,71^. Studies have shown that PsbR is necessary to maintain the conformation of PSII complex and stabilize the binding of PsbP and PsbQ^66,68,70^. Therefore, knocking out *psbR* reduces the rate of oxygen release and the reoxidation of quinones, which, in turn, affects photosynthetic efficiency^66,68,71^. PS I consists of at least 13 subunits. One of the most interesting low molecular weight (LMW) proteins associated with PSII is the PsbW subunit, a 6.1 kDa protein that was originally described as an intrinsic component of PSII in spinach (*Spinacea oleracea*)^72,73^. PsbW binds to the Lhcb proteins in the later steps of PSII assembly^74^, and its primary location is in the PSII-LHCII super complex^75,76^. Cyt b_6f._ is involved in electron transfer. The Rieske Fe/S protein has been isolated from plant cytochrome b_6f._ complexes, such as spinach^77^ and pea (*Pisum sativum*)^78^, and it is known that the protein is encoded by the nuclear gene *PetC*^79^. In experiments in which rice^80^ and *Arabidopsis thaliana*^81^ were transformed with the *PetC* gene, the PetC mature protein was found to be enriched in the leaves, which increased the electron transfer capacity of photosynthetic system and thus, increased the yield. The top enriched KEGG item in the leaves of *H. beccarii* were related to photosynthesis (Fig. 6 and Table S7). The rich set of photosynthetic genes (Table S7) includes the genes that encode the oxygen-evolving complex (*PsbR*, *PsbO*, *PsbP* and *PsbQ*), photosynthetic system I (*PsaD*, *PsaE*, *PsaF*, *PsaH*, *PsaK*, *PsaL* and *PsaN*), PSII-LHCII supercomplexes (*PsbW*) and the cytochrome b_6f._ complex (Rieske [Fe-S] protein). This is consistent with the fact that leaves are the primary organs for photosynthesis. Some seagrasses, such as *P. oceanica*, have genes related to photosynthesis in their female flowers^13^. In fact, in *Posidonia* species, seeds and green fruits may also undergo photosynthesis^82^. Female flowers rather than male flowers have photosynthetic activity in *Posidonia*. Otherwise, the lack of this "additional" resource supply and significant investment in sexual reproduction of the species could pose a risk to the survival of these important flowering plants.

In conclusion, we obtained a high-quality *H. beccarii* transcriptome using a PacBio SMRT sequencing platform. The results are of great value to further annotate the genome of *H. beccarii* and optimize its gene structure. In addition, these findings can provide important information for the future study of gene functions in this species.

## Materials and methods

### Sample collection and RNA preparation

The plant materials of *H. beccarii* were collected in Shajing, Qinzhou, Guangxi, China (21° 84′ 56.08′′ N, 108° 57′ 34.88′′ E) on November 5, 2021. The sampling site, outside mangrove forests, was covered by dense *H. beccarii*. The leaf and rhizome tissues were washed with ultrapure water, dissected, immediately frozen in liquid nitrogen, and stored at −80 °C.

We obtained permission from the Beilun Estuary Preserve in Guangxi to collect the samples, which were collected in compliance with the Convention on the Trade in Endangered Species of Wild Fauna and Flora (https://www.cites.org/). The formal species was identified by Guanglong Qiu (Guangxi Mangrove Research Center), and voucher specimens (GMRCHC081) were deposited in the Guangxi Mangrove Research Center.

In particular, the leaf and rhizome tissue samples were mixed equally to extract the total RNA to generate a pool to construct a SMRT library of *H. beccarii*. Total RNA was extracted from each tissue for Illumina sequencing (all six samples, two tissues, and three biological replicates) using an EasySpin Plant RNA Rapid Extraction kit (RN40, Aidlab) according to the manufacturer’s instructions and then treated with RNase-free DNase I (TianGen, Beijing, China) to remove the genomic DNA. High quality RNA is the basis of successful sequencing. To ensure the accuracy of sequencing data, we used the following methods to test the samples, and the libraries were only constructed after the test results met the requirements. A Nanodrop spectrophotometer was used to test whether the purity (A_260_/A_280_), concentration, and nucleic acid absorption peak of the RNA were normal. An Agilent 2100 accurately detects the integrity of RNA. The detection indicators include the RNA integrity number (RIN) value, 28S/18S, whether the baseline of the map is lifted or not, and the 5S peak. An electrophoretic analysis indicates whether the RNA samples are contaminated with genomic DNA. High-quality RNA samples with RIN ≥ 8.0 were used to construct the cDNA library for PacBio sequencing.

### Library construction, SMRT sequencing, and quality control

First-strand cDNA was synthesized using a SMARTer PCR cDNA Synthesis Kit (Clontech, Mountain View, CA, USA). PCR amplification and enrichment were conducted with reverse transcription cDNA as the template, and the amplified products were purified and recovered with 0.8 X AMpure PB magnetic beads (Beckman Coulter, Pasadena, CA, USA). The concentration (Qubit) and size (Agilent 2100) of the purified product were detected, and equimolar mass mixing was conducted based on the fragment size. A SMRTbell Template Prep Kit provided by PacBio was used to repair damage, repair the ends and connect the joint of the mixed product. The reactions were performed on a PCR instrument or in a constant temperature water bath. One SMRTbell Template library was then constructed and sequenced with the PacBio Sequel platform.

### SMRT sequencing data processing

The raw reads were processed into CCS reads using the PacBio SMRT analysis software v2.3.0 (http://www.pacb.com/products-andservices/analytical-software/smrt-analysis/) to remove low-quality polymerase reads, which utilized the threshold of read length < 50 bp and read score < 0.75. An FLNC sequence is a type of full-length non-chimeric CCS that meets the primers at both ends. The poly-A tail at the 3' end is completely sequenced, and there is no sequence chimerism. We adopted two strategies to ensure that the FNLC was accurately corrected. The first was self-correction using the Iterative Clustering and Error Correction (ICE) tool of the cluster module of SMRT Link software to cluster and correct multiple highly similar FLNC sequences to obtain the non-redundant FLNC sequences. The non-full-length non-chimeric sequences are filtered out when generating the FLNC, which further corrects the redundant FLNC sequences and improves the sequence quality. The second strategy was to align the RNA-Seq data based on the second-generation sequencing platform to the FLNC sequence to complete the correction, which was completed by proofread software. Finally, the cd-hit program was used to merge the high-quality full-length transcripts obtained by the two strategies to remove redundancy and finally obtain high-quality nonredundant full-length transcripts for subsequent analysis.

### ORF and the prediction of TFs, functional annotation, predictions of lncRNAs and SSRs and reference sequence alignment

The ORFs were identified via TransDecoder software (-m 100 -S). The TFs were identified based on plantTFDB 5.0^83^ using the diamond BLASTP program (evalue < 1e^−5^, min_cov > 40%). SWISS-PROT, Pfam, KEGG, GO, nr, KOG and TrEMBL were used to annotate the full-length transcripts using the diamond BLASTP program (evalue < 1e^−5^, min_cov > 40%). lncRNA candidates were screened with the threshold that the transcripts were longer than 200 nt by combing PLEK (-minlength 200), CPC2.0 (-r FALSE), CPAT (-s ATG -t TAA, TAG, TGA) and Pfam. The Pfam_scan.pl program was used to annotate the Pfam database (Pfam_A). The SSR sites in the transcripts were predicted through the misa.pl program (misa.ini–definition 1–10 2–6 3–5 4–5 5–5 6–5–interruptions 100). We used hisat2 software to compare the original sequencing reads to the full-length transcript to quantify the subsequent level of gene transcription, reconstruct the transcript and discover new genes (-q –phred33 –sensitive).

### Analysis of DEGs

The R language package DESeq2 (http://www.bioconductor.org/packages/release/bioc/html/DESeq2.html) was used to analyze the differential gene expression. The screening threshold was false discovery rate (FDR) < 0.05, log_2_FC (fold change (rhizomes/leaves) for a gene) > 1 or log_2_FC < −1.

### KEGG enrichment analysis of the DEGs

Pathway significance enrichment analysis used the KEGG pathway as the unit and applied a hypergeometric test to locate the pathways that were significantly enriched in differential genes compared with all the annotated genes. A path with FDR ≤ 0.05 is defined as a path that is significantly enriched in DEGs. R software (https://cran.r-project.org/; version 3.4.4), combined with self-writing scripts, was used to establish the parameter—FDR as BH (i.e., using BH correction) for a path enrichment analysis. The differential genes, upregulated genes, and downregulated genes were enriched and analyzed using KEGG^84,85^.

## Supplementary Information

Supplementary Information 1.

Supplementary Information 2.

Supplementary Information 3.

Supplementary Information 4.

Supplementary Information 5.

Supplementary Information 6.

Supplementary Information 7.

Supplementary Information 8.

Supplementary Information 9.

Supplementary Information 10.

## Data Availability

Data generated or analyzed during this study are included in this published article and its supplementary information files. PacBio SMRT reads and Illumina SGS reads generated in this study have been submitted to the BioProject database of National Center for Biotechnology Information (accession number PRJNA823762, http://www.ncbi.nlm.nih.gov).
