# Supplementary material for: Single-molecule real-time sequencing of the full-length transcriptome of Halophila beccarii
Source: Sci Rep. 2022 Sep 30;12:16444. doi: 10.1038/s41598-022-20988-w (PMC9525579; doi:10.1038/s41598-022-20988-w)
Supplement: Supplementary file 1 — Supplementary Information 1. [file 41598_2022_20988_MOESM1_ESM.pdf]

Supplementary Information

Scientific Reports

Single-molecule real-time sequencing of the full-length transcriptome of *Halophila beccarii*

Siting Chen[1*], Guanglong Qiu[1*]

[1]Guangxi Key Lab of Mangrove Conservation and Utilization, Guangxi Mangrove Research Center, Guangxi Academy of Sciences, Beihai, Guangxi, 536007, China

[*]Corresponding author at: Guangxi Key Lab of Mangrove Conservation and Utilization, Guangxi Mangrove Research Center, Guangxi Academy of Sciences, Beihai, Guangxi, 536007, China

E-mail address: c105043041@126.com; gqiu@mangrove.org.cn

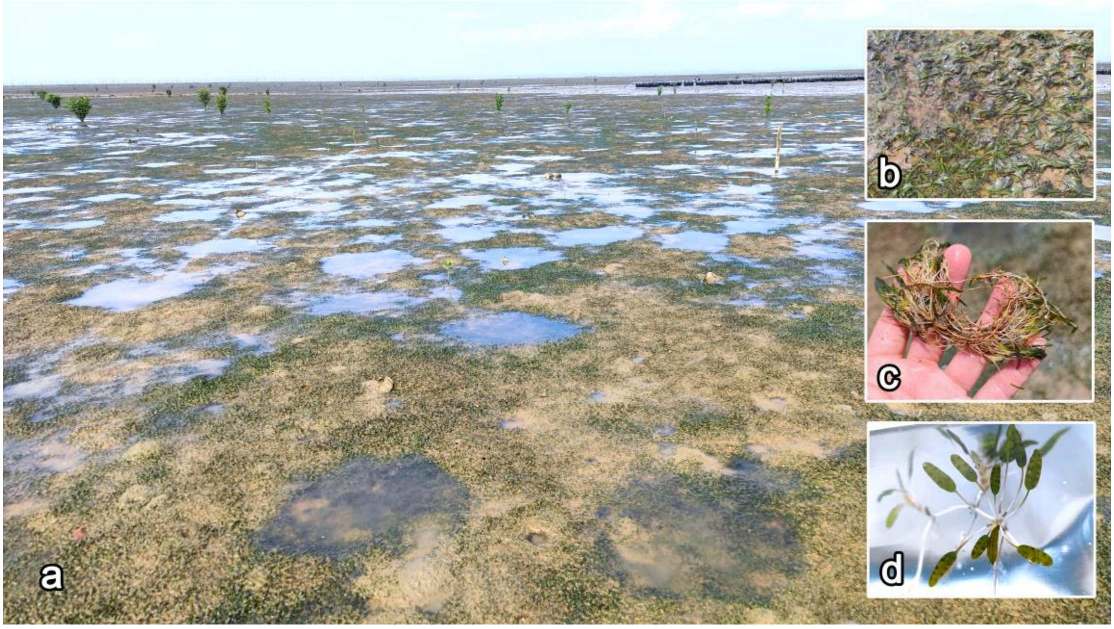

Fig. S1 *Halophila beccarii* and its' growing habitat. (**a**) An intertidal growing habitat. (**b**) Seagrass community. (**c**) Dug out seagrass plants with rhizomes and roots. (**d**) 5+ elongated oval leaves arranged in clusters

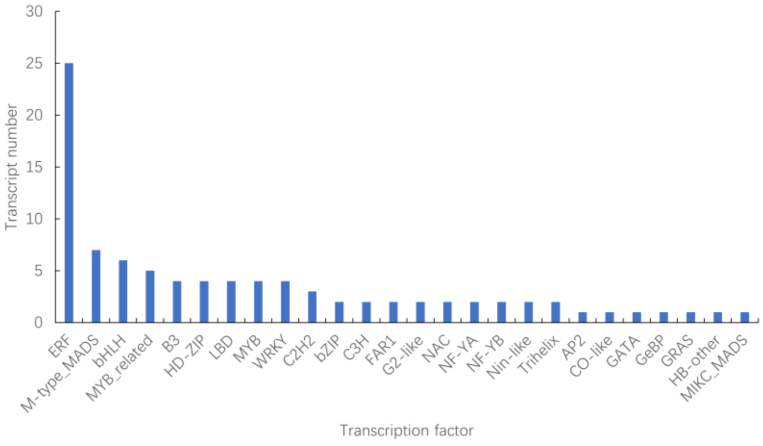

Fig. S2 Type distribution of TFs in differentially expressed genes
