# Supplementary material for: Single-molecule real-time sequencing of the full-length transcriptome of Halophila beccarii
Source: Sci Rep. 2022 Sep 30;12:16444. doi: 10.1038/s41598-022-20988-w (PMC9525579; doi:10.1038/s41598-022-20988-w)
Supplement: Supplementary file 10 — Supplementary Information 10. [file 41598_2022_20988_MOESM10_ESM.docx]

**Legends for Supplementary Tables and Figures**

**Table S1** Functional annotation of identified transcripts

**Table S2** lncRNAs detected by PLEK, CPC2.0, CPAT and Pfam

**Table S3** Statistical analysis of SSRs

**Table S4** Predicted SSRs and corresponding primers

**Table S5** Alignment ratio between reads and full-length transcripts

**Table S6** Differential gene expression analysis

**Table S7** KEGG enrichment analysis of differentially expressed genes

**Table S8** The rich set of photosynthetic genes

**Fig. S1** *Halophila beccarii* and its' growing habitat. (**a**) An intertidal growing habitat. (**b**) Seagrass community. (**c**) Dug out seagrass plants with rhizomes and roots. (**d**) 5+ elongated oval leaves arranged in clusters

**Fig. S2** Type distribution of TFs in differentially expressed genes
